# Inducible Knock-Down of the Mineralocorticoid Receptor in Mice Disturbs Regulation of the Renin-Angiotensin-Aldosterone System and Attenuates Heart Failure Induced by Pressure Overload

**DOI:** 10.1371/journal.pone.0143954

**Published:** 2015-11-25

**Authors:** Elena Montes-Cobos, Xiao Li, Henrike J. Fischer, André Sasse, Sebastian Kügler, Michael Didié, Karl Toischer, Martin Fassnacht, Ralf Dressel, Holger M. Reichardt

**Affiliations:** 1 Institute for Cellular and Molecular Immunology, University Medical Center Göttingen, 37073 Göttingen, Germany; 2 Department of Neurology, Center for Molecular Physiology of the Brain, University Medical Center Göttingen, 37073 Göttingen, Germany; 3 Institute of Pharmacology, University Medical Center Göttingen, 37073 Göttingen, Germany; 4 Department of Cardiology and Pneumology, University Medical Center Göttingen, 37073 Göttingen, Germany; 5 Endocrine and Diabetes Unit, Department of Internal Medicine I, University of Würzburg, 97080 Würzburg, Germany; 6 Partner site Göttingen, German Center for Cardiovascular Research (DZHK), 37073 Göttingen, Germany; INSERM, FRANCE

## Abstract

Mineralocorticoid receptor (MR) inactivation in mice results in early postnatal lethality. Therefore we generated mice in which MR expression can be silenced during adulthood by administration of doxycycline (Dox). Using a lentiviral approach, we obtained two lines of transgenic mice harboring a construct that allows for regulatable MR inactivation by RNAi and concomitant expression of eGFP. MR mRNA levels in heart and kidney of inducible MR knock-down mice were unaltered in the absence of Dox, confirming the tightness of the system. In contrast, two weeks after Dox administration MR expression was significantly diminished in a variety of tissues. In the kidney, this resulted in lower mRNA levels of selected target genes, which was accompanied by strongly increased serum aldosterone and plasma renin levels as well as by elevated sodium excretion. In the healthy heart, gene expression and the amount of collagen were unchanged despite MR levels being significantly reduced. After transverse aortic constriction, however, cardiac hypertrophy and progressive heart failure were attenuated by MR silencing, fibrosis was unaffected and mRNA levels of a subset of genes reduced. Taken together, we believe that this mouse model is a useful tool to investigate the role of the MR in pathophysiological processes.

## Introduction

The mineralocorticoid receptor (MR) is a member of the nuclear receptor superfamily and involved in mediating the organism’s response to aldosterone and glucocorticoid hormones (GCs) such as corticosterone and cortisol [1]. After ligand binding, the MR translocates into the nucleus where it acts as a transcription factor. Although the MR is able to bind GCs, its main ligand in kidney and colon is aldosterone. The reason is that 11β-hydroxysteroid dehydrogenase type 2 present in these organs inactivates GCs, thereby resulting in the exclusive occupation of the MR by aldosterone [2]. In contrast, no such mechanism exists in neurons, macrophages and cardiomyocytes. Hence, in these cell types GCs are the predominant ligand of the MR and responsible for most of its activities.

The MR plays a central role in the regulation of salt-water homeostasis mediated by the renin-angiotensin-aldosterone system (RAAS) [2]. In the kidney, aldosterone induces sodium reabsorption via the MR, which serves to regulate extracellular fluid volume and contributes to blood pressure control [3]. This effect is primarily achieved by upregulating transport proteins such as the amiloride-sensitive epithelial sodium channel (ENaC). Hence, MR-deficient mice postnatally showed clinical symptoms that are reminiscent of pseudohypoaldosteronism type I, which is characterized by increased plasma renin activity [4]. MR knock-down rats had a similar albeit milder phenotype, thus mimicking the partial loss of MR function occasionally encountered in patients [5]. Noteworthy, mice with a constitutive or an inducible renal principal cell-specific MR disruption had elevated levels of aldosterone, whereas ENaC activity and sodium excretion were normal [6, 7].

Cardiomyocytes are another important site of MR expression, and antagonists such as spironolactone and epelerone are believed to mainly displace GCs bound to the MR in this cell type and thereby improve the outcome of patients suffering from heart diseases [8]. Accordingly, disruption of the MR in cardiomyocytes enhanced infarct healing and attenuated cardiac failure although it had no impact on heart function under basal conditions [9]. Moreover, these knock-out mice were also protected from cardiac failure in a model of increased afterload, reconfirming that the MR in cardiomyocytes mediates detrimental effects in the heart under pathological conditions [10].

The MR expressed by macrophages has recently gained attention as well because it is involved in mediating the effects of GCs on the inflammatory phenotype of myeloid cells [11, 12]. Due to the involvement of macrophages in heart physiology, MR ablation in this cell type protected against hypertrophy, fibrosis, vascular damage and progressive heart failure [13, 14]. The MR is also present in smooth muscle cells and thereby contributes to blood pressure control. Consequently, cell type-specific disruption in mice resulted in hypotension and a decreased vascular tone [15]. Finally, the MR is found in hippocampus and amygdala, where it is involved in the modulation of cognitive processes such as learning and memory [16].

The first generation of knock-out mice by homologous recombination in embryonic stem cells and the development of the Cre-loxP technique revolutionized medical research [17, 18]. An alternative technique to inactivate gene function in a wide range of species is RNA interference (RNAi) [19]. Several years ago, this biological process has been developed into an experimental tool which allows to stably silence genes in cells and transgenic animals by expression of small hairpin RNAs (shRNA) [20]. This is often achieved by using lentiviruses since they are able to infect non-dividing cells. Furthermore, there is the possibility to confer temporal control to the system by modifying the promoter that drives shRNA expression [21]. Here we report the generation of transgenic mice in which MR expression can be inducibly silenced, and their initial characterization with regard to pathophysiological consequences in kidney and heart.

## Material and Methods

### Cloning of the lentiviral vector

An optimized lentiviral vector for inducible shRNA expression was cloned on the basis of a previously reported set of two plasmids [21]. To this end, the BbsI site in the pH1tet-flex vector was replaced by an AgeI site. Subsequently, the Sall-Xhol fragment was excised and inserted into the Xhol restriction site of the FUTG vector. The resulting construct allows for one-step cloning of any shRNA sequence to be expressed under the control of the inducible H1 promoter. Using this vector, five shRNA sequences specific for the MR were tested for their ability to reduce its expression in cell culture, and the most efficient one was selected for the generation of transgenic mice. The sequences of the sense and antisense oligonucleotides used for cloning of the chosen vector are the following ones: 5’-CCGGGCTC-TACTTTACGAAGTGTTT-TTCAAGAGA-AAACACTTCGTAAAGTAGAGC-TTTTTC-3’ and 5’-TCGAG-AAAAA-GCTCTACTTTACGAAGTGTTT-TCTCTTGAA-AAACACTTCGTAAAGTAGAGC-3’.

### Production of lentiviral particles

VSV-G pseudotyped lentiviral particles were produced in 293T cells (3 x 10^8^ cells per production) in 4-level cell factories (Nunc, Langenselbold, Germany). They were collected from the serum-free cell culture supernatant 48 hrs after transfection, filtered through a 0.22 μm cartridge (Merck Millipore, Schwalbach, Germany), and precipitated by two rounds of ultracentrifugation through a 30% sucrose cushion. The lentiviral particles were carefully re-suspended in a final volume of 50–75 μl PBS, which resulted in titers of 0.5–1.0 x 10^10^ infective particles per ml, and used for microinjection into mouse oocytes.

### Generation of transgenic mice

Transgenic mice were generated according to our standard protocols [22, 23]. In brief, C57BL/6 oocytes were microinjected with the highly concentrated lentivirus solution into the perivitellin space and cultured overnight. On the following day, two-cell stage embryos were transferred into the oviduct of pseudo-pregnant CD1 females. Transgenic offspring was identified by flow cytometric analysis of eGFP expression in peripheral blood leukocytes and backcrossed to wildtype C57Bl/6 mice for more than 5 generations. All experiments were approved by the responsible authority of Lower Saxony (*Nds*. *Landesamt für Lebensmittelsicherheit und Verbraucherschutz*, *Az*: *33*.*9-42502-04-12/0990 and 33*.*9-42502-04-11/0702*) and conducted in accordance to the ethical standards of humane animal care.

### Animal experimentation

To achieve MR silencing *in vivo*, doxycycline (Dox) was administered by offering food pellets containing 735 mg/kg doxycycline hyclate (Ssniff Spezialdiäten, Soest, Germany; effective concentration: 625 mg/kg Dox) *ad libitum* to the mice for at least 14 days. Control animals received a standard diet from the same supplier. Blood was collected by heart puncture after having sacrificed the mice, and serum and plasma were separated using Microtainer SST or K2E tubes, respectively (BD Biosciences, Heidelberg, Germany). In order to perform gene expression and histological analyses, individual organs were dissected and either snap-frozen in liquid nitrogen or fixed in formaldehyde solution.

### Flow cytometry

Blood was collected from the tail tip of the mice into Alsever’s solution and subjected to erythrolysis with Optilyse^™^ (Immunotech, Marseille, France) according to the manufacturer´s instructions. Flow cytometric analysis was employed to determine eGFP expression in peripheral blood leukocytes using a FACSCanto II device (BD Biosciences) in combination with FlowJo software (Tree Star, Ashland, OR).

### RNA isolation, cDNA synthesis and quantitative RT-PCR analysis

Frozen tissue was homogenized using an Ultra-Turrax disperser (IKA-Werke, Germany) and RNA extraction was performed with the help of the RNeasy plus Universal Kit (Qiagen, Hilden, Germany) according to the manufacturer´s instructions. 1 μg RNA was reverse transcribed with the iScript^™^ Reaction Mix kit (Bio-Rad, Munich, Germany) and quantitative RT-PCR (RT-QPCR) was performed using the Power SYBR^®^ Mix (Applied Biosystems, Darmstadt, Germany). Results were normalized to the expression of the house-keeping gene HPRT and evaluated using the ΔΔCt method. Primer sequences are available upon request.

### Hormone and electrolyte measurements

Aldosterone levels in the serum were measured by RIA (IM1664, Immunotech Beckman Coulter, Prague, Czech Republic), and renin levels in the plasma were determined using a mouse-specific ELISA (ELM-Renin1, RayBiotech, Norcross, GA). Both assays were performed according to the manufacturers’ instructions using appropriately diluted samples. Na^+^ and K^+^ concentrations in the urine were measured with ion selective electrodes as reported earlier [5]. Blood and urine samples were always collected in the morning.

### Western blot analysis

Lysates were prepared in denaturing sample buffer containing a protease inhibitor cocktail using a Dounce homogenizer and heated at 95°C for 5 min. Proteins were separated on a SDS-PAGE gel, transferred to a nitrocellulose membrane (Amersham, Braunschweig, Germany) and stained with an anti-MR (Santa Cruz, Heidelberg, Germany) or an anti-ERK (Santa Cruz) antibody. Visualization was achieved with a ChemiLux Imager (Intas, Göttingen, Germany) and ECL substrate (Carl Roth, Karlsruhe, Germany). Densitometric quantification of the band intensities was achieved using GelPro analyzing software (Media Cybernetics, Rockville, MD); the specific background was individually subtracted in each case.

### Histology and immunohistochemistry

Hearts were perfused *ex vivo* via the aorta with 0.9% NaCl before fixation in 3.7% formaldehyde solution overnight. After embedding in paraffin, 5 μm sections were cut and stained for 60 min at room temperature in Sirius Red solution, i.e. 0.1% Direct Red 80 (Sigma, Taufkirchen, Germany) in saturated aqueous picric acid (Morphisto, Frankfurt, Germany) adjusted to pH 2.0 with sodium hydroxide [24]. The slides were tipped four times in 0.5% acetic acid solution and incubated 5 min each in ethanol and in isopropyl alcohol. After two further incubations for 10 min in xylole, the slices were embedded in Histokitt (Carl Roth) and recorded at 20x magnification using a slide scanner (Olympus, Hamburg, Germany). The extent of fibrosis based on the amount of collagen was quantified in two complete heart sections using *cellSens Dimensions* software (Olympus) by a scientist blinded to the genotype of the mice.

Kidneys were immersion-fixed in 3.7% formaldehyde solution overnight and embedded in paraffin. Immunohistochemical demonstration of eGFP was performed on 5 μm sections using a rabbit anti-eGFP polyclonal antibody (Abcam, Cambridge, UK) and the Vectastin ABC kit (Vector Laboratories, Burlingame, CA) using DAB (3,3’-diaminobenzidine tetrachloride) as a substrate. Quenching of endogenous peroxidase activity was achieved by treating the sections with 3% H_2_O_2_ in ethanol.

### Transverse aortic constriction (TAC)

TAC surgery was done using a minimally invasive operation procedure as described previously [25]. 11 to 12 weeks old mice were anesthetized using intraperitoneal injections of medetomidin (0.5 mg/kg), midazolam (5 mg/kg), and fentanyl (0.05 mg/kg). After horizontal incision at the jugulum the transversal aorta was displayed and a 26 gauge needle was tied against the aorta. Then the needle was removed, the skin was closed and the anesthesia was reversed by subcutaneous injection of atipamezol (2.5 mg/kg) and flumazenil (0.5 mg/kg). The mice were kept on a heating plate until recovering from anesthesia and received subcutaneously buprenorphin (60 μg/kg) for further analgesia 1 hr after surgery. For long-term analgesia metamizole (1.33 mg/ml) was added to the drinking water. Three days after surgery, the pressure gradient over the aortic ligature was determined using pulsed wave Doppler. At the end of the experiment the mice were sacrificed in isofluran anesthesia (5%) by cervical dislocation. The hearts were excised, perfused with saline solution and after weighting of the ventricles immediately processed for RNA isolation or fixation.

### Transthoracic Echocardiography

Transthoracic echocardiography was performed blinded using a Vevo2100 (VisualSonics, Toronto, Canada) system with a 30 MHz center frequency transducer. The animals were anesthetized with 3% isoflurane, and temperature, respiration, and ECG-controlled anesthesia was maintained with 1.5% isoflurane [25]. B-mode recordings of a modified parasternal short and long axis view at the midpapillary level were used to determine the long axis diameter in systole (Ls) and diastole (Ld), the end-diastolic (LVIDd) and end-systolic (LVIDs) left ventricular (LV) chamber diameter and the anterior (AWTh) and posterior wall thickness (PWTh), the area of the endocardium in systole (area s) and diastole (area d) and the area of the epicardium in systole (Epi s). The evaluation was done blinded to the genotype of the mice. Fractional area shortening (FAS) was calculated as (area d—area s) / area d x 100. Ejection fraction was calculated as ((5/6) x area d x Ld))—((5/6) x area s x Ls) / ((5/6) x area d x Ld) x 100. Echocardiographic LV weight (LVW) was estimated using the formula: 1.05 g/cm^2^ x (5/6) x (Epi s x (Ls + (AWTh + PWTh) / 2))–(area s x Ls).

### Statistical analysis

Data was analyzed using GraphPad Prism software (San Diego, California, USA). Data is depicted as mean values ± SEM. The unpaired two-tailed Student’s t test was used in all cases; p values above 0.05 were considered as non-significant (n.s.); *: p <0.05; **: p <0.01; and ***: p <0.001.

## Results

### Generation of inducible MR knock-down mice

**Fig 1:**
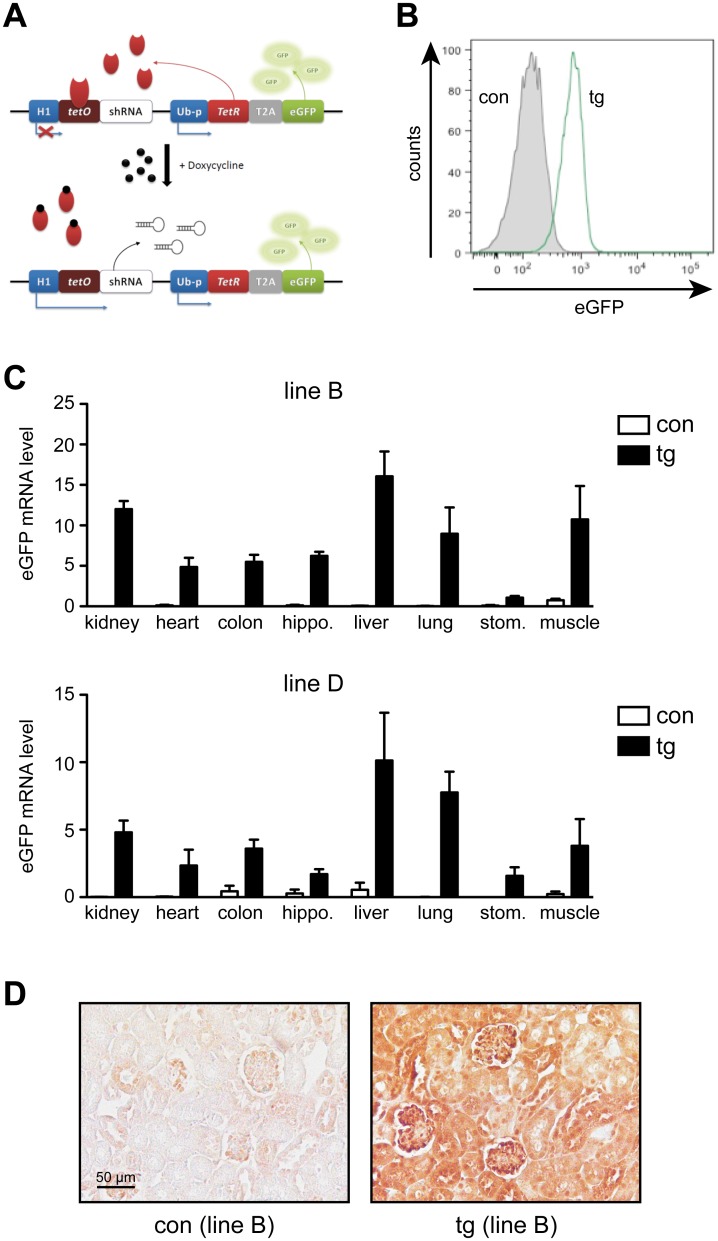
Generation and characterization of inducible MR knock-down mice. (A) Operating principle of the lentiviral system used for inducible MR knock-down. The vector comprises one cassette consisting of the H1 promoter with tetracycline resistance operator sequences (tetO) and a MR-specific shRNA, and a second one encompassing the tet repressor (TetR) linked to eGFP by a T2A element under the control of the ubiquitin C promoter (Ub-p). In the absence of doxycycline (Dox), the TetR binds to tetO and blocks shRNA expression. After addition of Dox, the TetR is released thus enabling shRNA transcription. Under both conditions, eGFP is expressed constitutively. (B) Peripheral blood leukocytes of one control (con) and one transgenic (tg) mouse were analyzed for eGFP expression by flow cytometry. (C) Kidney, heart, colon, hippocampus, liver, lung, stomach and muscle samples obtained from control (con) and transgenic (tg) mice of line B and D were analyzed for eGFP mRNA expression by RT-QPCR. N = 3–7 (line B), N = 3–5 (line D). Gene expression was normalized to HPRT and is depicted in arbitrary units as mean ± SEM. (D) Kidney sections from one control (con) and one transgenic (tg) mouse of line B were analyzed by immunohistochemistry for eGFP expression. One representative example out of three is shown for each genotype. Size bar: 50 μm.

A lentiviral vector was cloned to enable inducible expression of a shRNA targeting the MR, accompanied by constitutive expression of eGFP (Fig 1A). This vector was used for the production of a highly concentrated lentiviral particle suspension, which was microinjected into the perivitellin space of fertilized oocytes from C57Bl/6 mice. Two-stage embryos were transferred into the oviduct of pseudo-pregnant female CD1 mice and several transgenic founders were identified based on eGFP expression in peripheral blood leukocytes (Fig 1B). The transgenic offspring were backcrossed to C57Bl/6 wildtype mice for more than five generations to ensure a single integration of the lentiviral vector. Using this strategy, we were able to derive two stable lines of inducible MR knock-down mice designated B and D, which were used for all further experiments.

RT-QPCR analysis was employed to monitor transgene expression across a variety of tissues. eGFP was detected in all tested organs of transgenic but not control mice, albeit at somewhat different levels (Fig 1C). The overall expression pattern, however, was comparable between both lines, suggesting that the observed differences were an intrinsic feature of the lentiviral construct. Immunohistochemistry was employed to investigate eGFP expression in the kidney. Positive staining was observed in the entire organ of transgenic mice whereas controls showed only faint background color (Fig 1D).

### Analysis of MR expression

**Fig 2:**
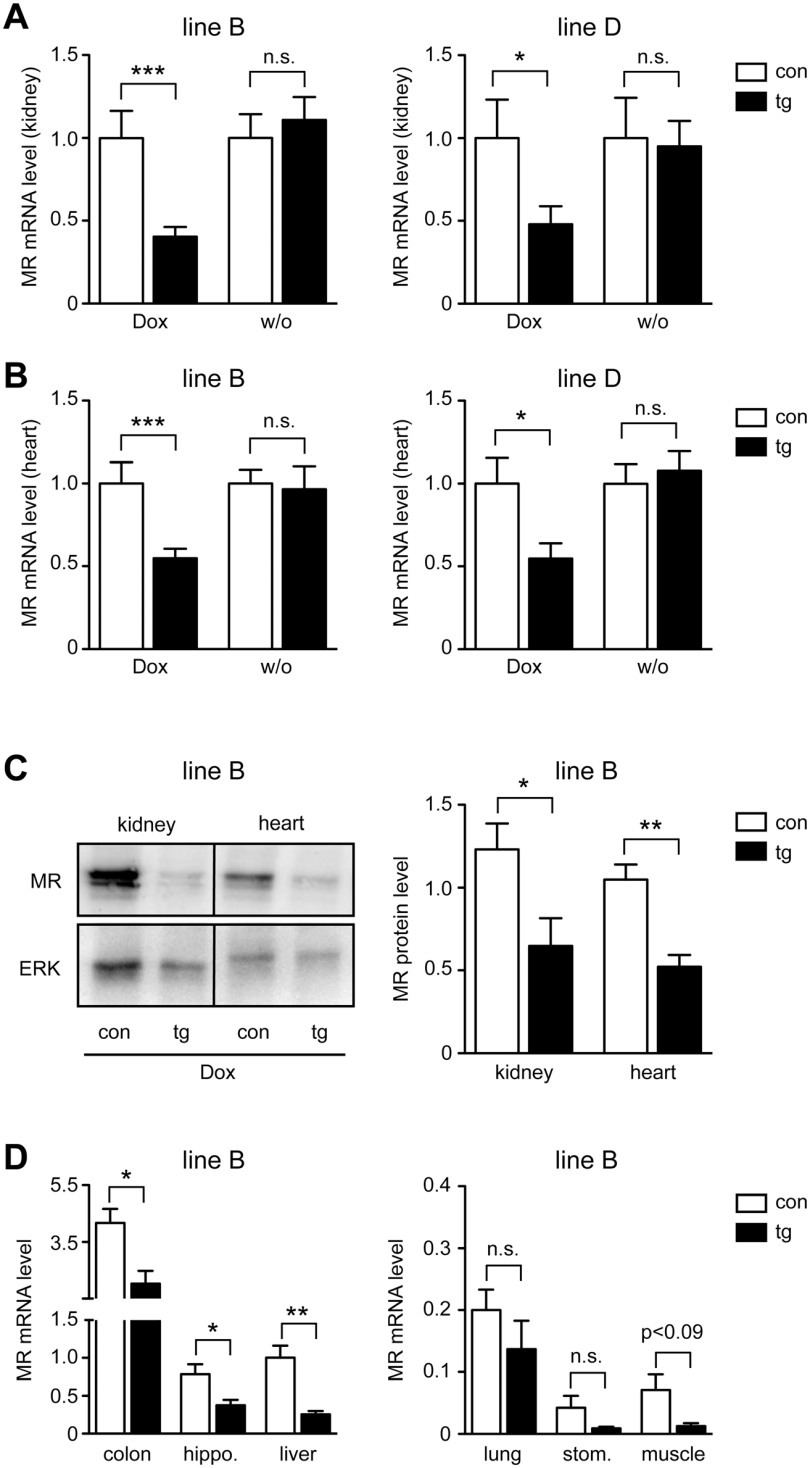
MR silencing in transgenic mice. Control (con) and transgenic (tg) mice of line B and D received Dox via food pellets for 14 days or, in some cases, were left untreated (w/o). (A) Whole kidneys were analyzed for MR mRNA expression by RT-QPCR. N = 14/22 (Dox, line B), N = 5/7 (w/o, line B), N = 13/20 (Dox, line D), N = 5/6 (w/o, line D). (B) Whole hearts were analyzed for MR mRNA expression by RT-QPCR. N = 9/17 (Dox, line B), N = 5/7 (w/o, line B), N = 13/14 (Dox, line D), N = 6/7 (w/o, line D). Gene expression in panels A and B was normalized to HPRT; mRNA levels in control mice were arbitrarily set to 1 and are depicted as mean ± SEM. (C) Protein lysates were prepared from kidney and heart of Dox-treated mice of line B and analyzed by western blot for the amount of MR protein. Detection of ERK served as a loading control. One representative analysis each is depicted in the left panel. The right panel shows a densitometric quantification of MR in kidney and heart of control (con) and transgenic (tg) mice after normalization to the amount of ERK. Protein levels are depicted in arbitrary units as mean ± SEM. N = 5/6. (D) Colon, hippocampus, liver, lung, stomach and muscle were isolated from Dox-treated mice of line B and analyzed for MR mRNA levels by RT-QPCR. Gene expression was normalized to HPRT and is depicted in arbitrary units as mean ± SEM. N = 4/7. Statistical analysis in all panels was performed by unpaired two-tailed Student’s t test. n.s.: non-significant; *: p <0.05; **: p <0.01; ***: p <0.001.

Transgenic and control mice of both lines received Dox via food pellets for two weeks or were left untreated. Subsequently, kidney and heart were isolated and RNA extracted from the entire organs. RT-QPCR analysis revealed that MR mRNA expression was significantly downregulated after Dox administration in both organs regardless of which line was analyzed (Fig 2A and 2B). In contrast, mRNA levels in untreated mice were unaltered, confirming that the inducible system was tight (Fig 2A and 2B). Western blot analysis confirmed that MR protein levels in kidney and heart (line B) were reduced to about the same extent as mRNA levels (Fig 2C). To obtain a more comprehensive picture of MR silencing we treated mice of line B for two weeks with Dox and studied a number of additional tissues by RT-QPCR. MR mRNA levels in hippocampus, liver and colon were significantly reduced in transgenic mice as compared to controls (Fig 2D). MR expression in stomach, muscle and lung was very low, but nevertheless, even in these organs we found a tendency to diminished mRNA levels in transgenic mice (Fig 2D).

### Gene expression analysis in kidney

**Fig 3:**
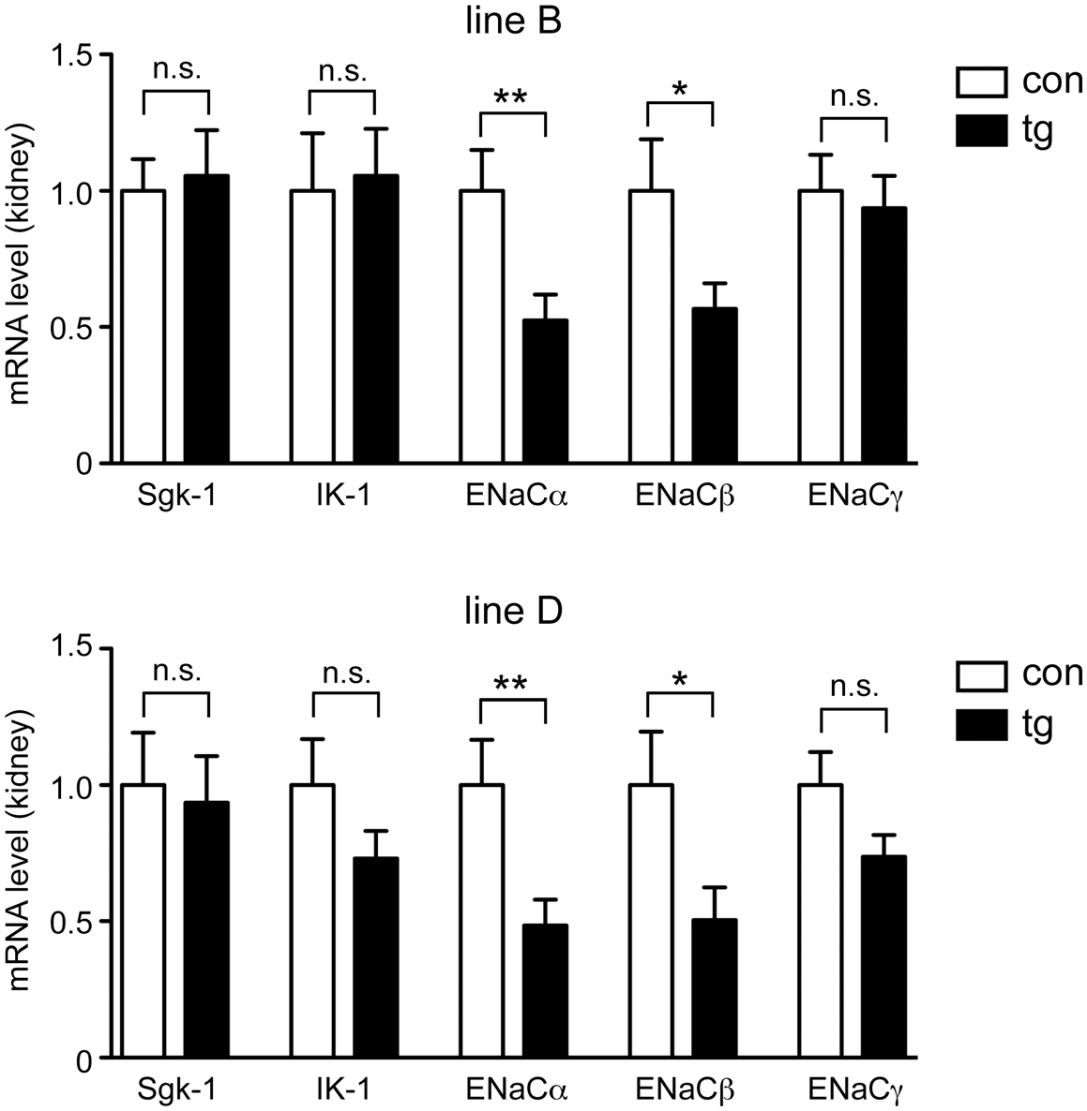
Gene expression analysis in the kidney of inducible MR knock-down mice. Control (con) and transgenic (tg) mice of line B (upper panel) and line D (lower panel) received Dox via food pellets for 14 days. Subsequently, whole kidneys were analyzed for mRNA expression of serum and glucocorticoid-regulated kinase 1 (Sgk-1), the potassium channel IK-1 and the subunits α, β and γ of the epithelial sodium channel ENaC by RT-QPCR. N = 16/24 (line B), N = 19/22 (line D). Gene expression was normalized to HPRT, and mRNA levels in control mice were arbitrarily set to 1. Values are depicted as mean ± SEM, statistical analysis was performed by unpaired two-tailed Student’s t test. n.s.: non-significant; *: p <0.05; **: p <0.01.

To study the impact of reduced MR expression in kidney, we initially determined mRNA levels of several putative MR target genes, namely serum and glucocorticoid-regulated kinase 1 (Sgk-1), the potassium channel IK-1, and the α, β and γ subunits of the epithelial sodium channel ENaC by RT-QPCR. Levels of ENaCα and β mRNA were significantly downregulated after Dox treatment in both lines of transgenic mice, whereas expression of the other three genes was unaltered (Fig 3).

### Serum aldosterone, plasma renin and urine electrolyte levels

**Fig 4:**
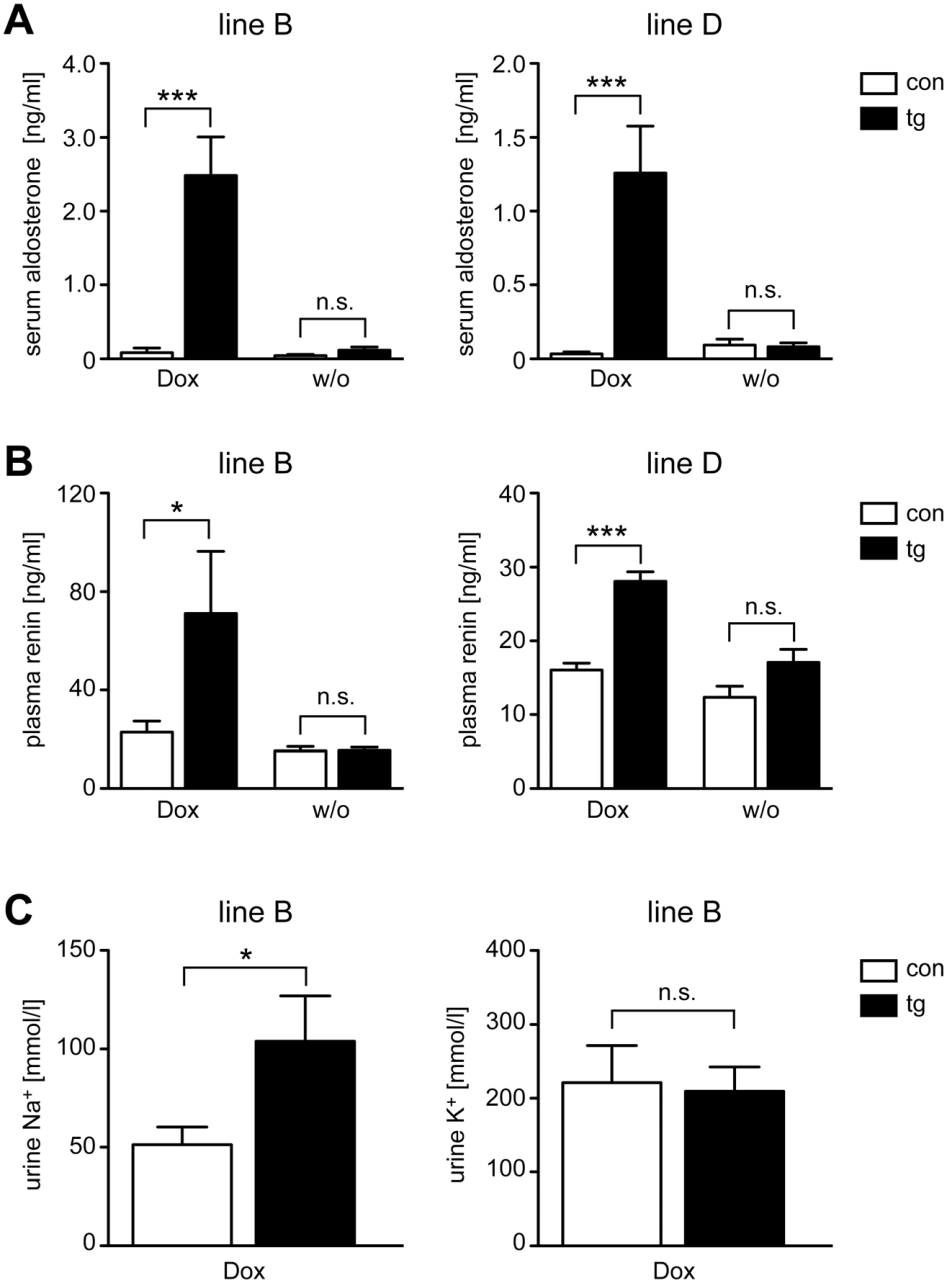
Regulation of the RAAS and salt excretion in inducible MR knock-down mice. Control (con) and transgenic (tg) mice of line B and D received Dox via food pellets for 14 days or were left untreated (w/o). Blood and urine samples were collected in the morning. (A) Serum was prepared and aldosterone levels were determined by RIA. N = 7/8 (Dox, line B), N = 7/7 (w/o, line B), N = 10/8 (Dox, line D), N = 6/8 (w/o, line D). (B) Plasma was prepared and renin levels were determined by ELISA. N = 12/8 (Dox, line B), N = 7/7 (w/o, line B), N = 9/7 (Dox, line D), N = 6/7 (w/o, line D). (C) Sodium (Na^+^) and potassium (K^+^) concentrations in the urine of mice of line B were measured using ion selective electrodes. N = 5/6. Values are depicted as mean ± SEM, statistical analysis was performed by unpaired two-tailed Student’s t test. n.s.: non-significant; *: p <0.05; ***: p <0.001.

To identify functional consequences of reduced MR expression in kidney, we tested the transgenic mice for pathophysiological changes in the regulation of the RAAS. Transgenic and control mice of both lines were either treated with Dox for two weeks or left untreated, followed by the analysis of serum aldosterone and plasma renin levels. Importantly, the concentration of aldosterone in the serum of transgenic mice treated with Dox was increased up to 1,000-fold (Fig 4A). A similar finding was made for plasma renin levels, which were also strongly elevated after Dox treatment (Fig 4B). In the absence of Dox, secretion of both hormones was largely unaffected (Fig 4A and 4B). Noteworthy, the increase in aldosterone and renin concentrations after administration of Dox was more pronounced in line B as compared to line D.

To investigate whether MR silencing affects sodium excretion, we measured electrolytes in the urine of mice of line B after treating them for two weeks with Dox. The concentration of sodium was significantly increased in the urine of transgenic mice compared to controls, whereas the concentration of potassium was unaltered (Fig 4C). This finding indicates that silencing of the MR leads to salt wasting.

### Analysis of gene expression and fibrosis in heart

**Fig 5:**
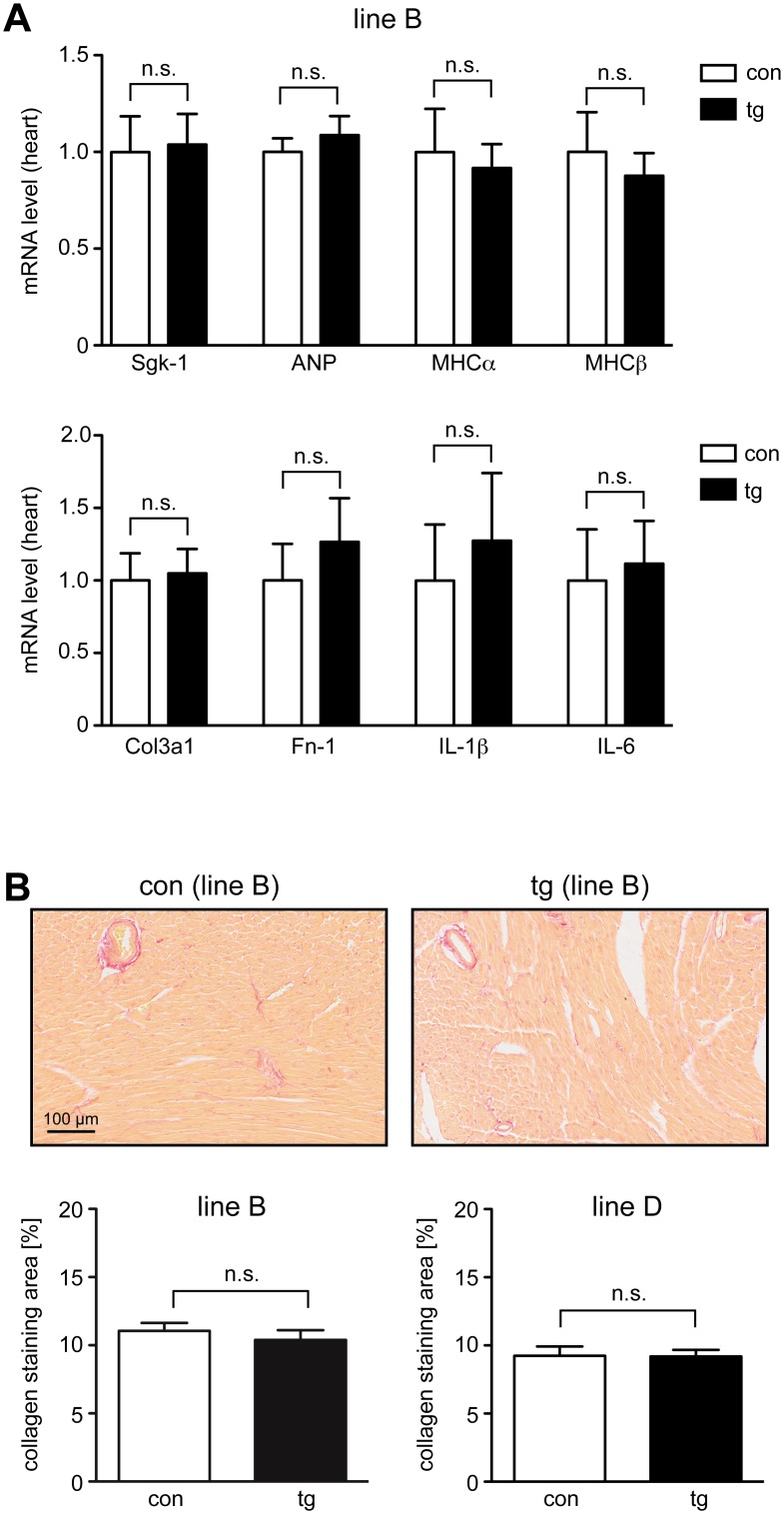
Gene expression analysis and collagen deposition in the heart of inducible MR knock-down mice. (A) Control (con) and transgenic (tg) mice of line B received Dox via food pellets for 14 days. In the upper panel, analysis of the heart for mRNA expression of serum and glucocorticoid-regulated kinase 1 (Sgk-1), the hormone ANP and the structural proteins MHCα and β by RT-QPCR is depicted. N = 10/17. In the lower panel, analysis of the heart for mRNA expression of Col3a1 (Collagen, type III, α1), fibronectin (FN-1) and the pro-inflammatory cytokines IL-1β and IL-6 by RT-QPCR is depicted. N = 3/4. Gene expression in panel A was normalized to HPRT; mRNA levels in control mice were arbitrarily set to 1 and are depicted as mean ± SEM. (B) Control (con) and transgenic (tg) mice of line B and D received Dox via food pellets for 28 days. Exemplary photographs of heart sections of one control and one transgenic mouse of line B, stained with picrosirius red are depicted in the upper panel. The red color corresponds to collagen mainly found around vessels. Size bar: 100 μm. The lower panel shows computer-aided quantification of the collagen staining area in control and transgenic mice of both line B and D. N = 5/5 (line B), N = 3/5 (line D). Values in panel B are depicted as mean ± SEM, statistical analysis was performed by unpaired two-tailed Student’s t test. n.s.: non-significant.

As we had observed that knock-down of the MR had a profound impact on gene expression in kidney and the regulation of the RAAS, we wondered whether the heart was affected as well. Initially, we studied gene expression of Sgk-1, the peptide hormone ANP and the structural proteins MHCα and β, but did not find any significant differences between Dox-treated transgenic and control mice of line B (Fig 5A). Furthermore, there were no differences with regard to the expression of the collagen component Col3a1 and fibronectin (Fn-1), two genes widely considered as markers of fibrosis, and in the expression of the pro-inflammatory cytokines IL-1β and IL-6 (Fig 5A).

To extend this study, we tested whether downregulation of the MR caused fibrosis in the healthy heart. To allow for sufficient time for tissue remodeling we treated transgenic and control mice with Dox for four weeks. Subsequently, they were perfused and the heart analyzed for collagen deposition using Picrosirius red staining (Fig 5B). Importantly, quantification of collagen did not reveal any signs of fibrosis in the heart of either transgenic mouse line (Fig 5B).

### Heart function under conditions of increased cardiac afterload

**Fig 6:**
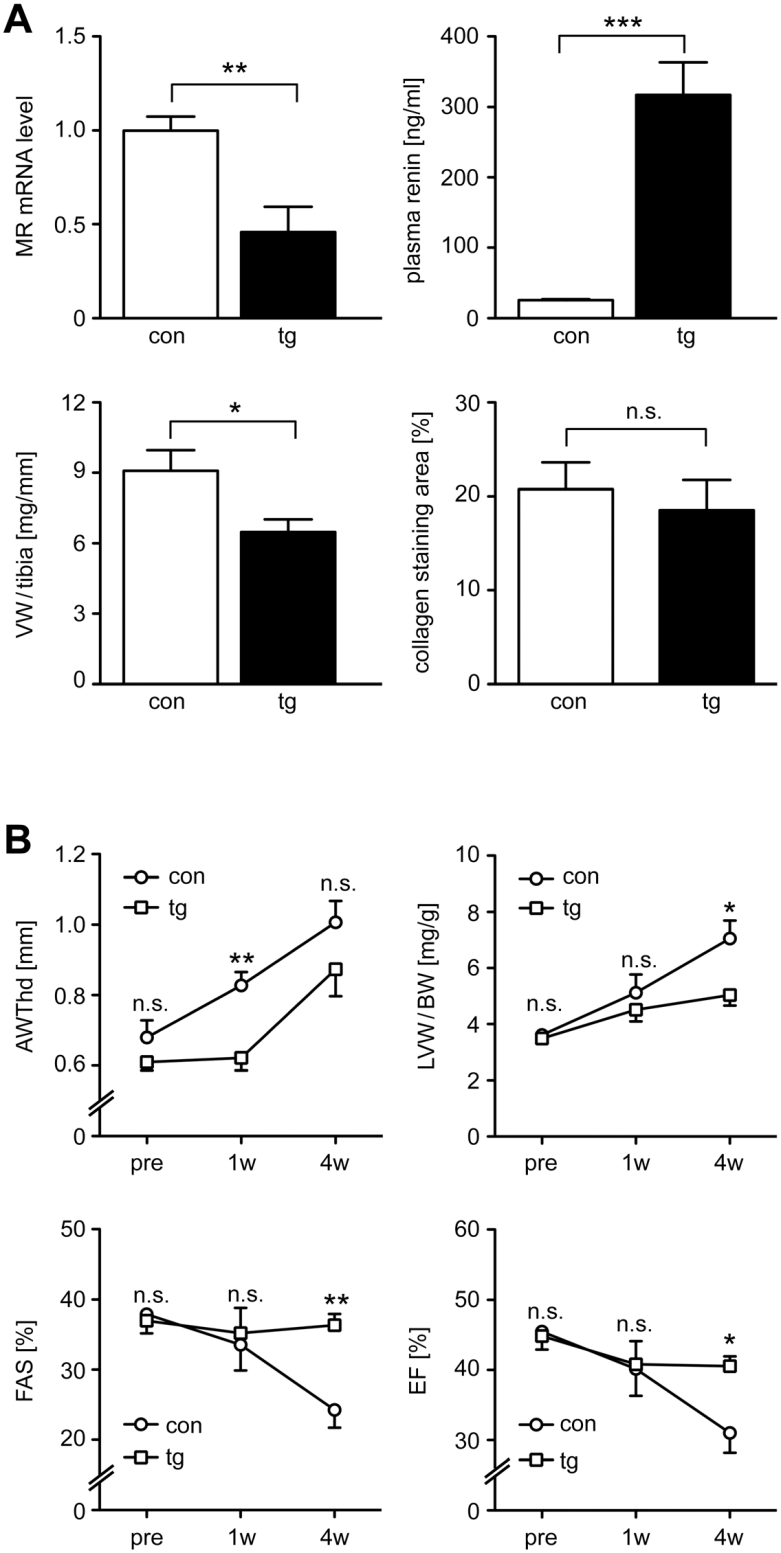
Physiological changes in inducible MR knock-down mice after TAC. (A) Control (con) and transgenic (tg) mice of line B received Dox via food pellets throughout the entire duration of the experiment. Two weeks after the beginning of Dox treatment TAC surgery was performed and another four weeks later the mice were sacrificed. MR mRNA levels in the heart were determined by RT-QPCR at the end of the experiment, renin plasma levels were analyzed by ELISA. The ventricular weight was measured for each animal and is depicted relative to the respective tibia lengths. The collagen staining area was determined after incubating paraffin sections from heart with picrosirius red followed by computer-aided quantification. (B) Analysis by echocardiography was performed one week before TAC (pre) as well as one (1w) and four weeks after TAC (4w). The diagrams show alterations of four parameters over time, namely the end-diastolic anterior wall thickness (AWThd), the left ventricular weight relative to the body weight (LVW/BW), the fractional area shortening (FAS) and the ejection fraction (EF). Values in all panels are depicted as mean ± SEM; N = 5/7. Statistical analysis was performed by unpaired two-tailed Student’s t test. n.s.: non-significant; *: p <0.05; **: p <0.01; ***: p <0.001.

Chronic pressure overload stress due to hypertension or aortic stenosis leads to progressive heart failure. However, treatment with MR antagonists as well as deletion of the MR in cardiomyocytes or macrophages prevent some of the pathogenic consequences [10, 14, 26]. It is against this background that we tested the impact of MR silencing on the response to transverse aortic constriction (TAC) as a model of increased cardiac afterload. Transgenic and control mice of line B were treated with Dox and two weeks later an echocardiography was performed followed by surgery. Analysis by pulsed wave Doppler three days after TAC revealed that the stenosis reached by this intervention was similar in transgenic and control mice (con: 49.5 ± 7.4 mmHg; tg: 45.4 ± 5.4 mmHg). Echocardiography was repeated one and four weeks after surgery, and at the end of the experiment heart and plasma samples were collected. RT-QPCR analysis indicated successful silencing of the MR, which was confirmed by the strong increase in plasma renin levels (Fig 6A). The relative ventricular weight (VW/tibia) in transgenic mice was significantly lower than in controls whereas fibrosis was similar in both genotypes (Fig 6A).

Echocardiography revealed that the anterior wall thickness (AWThd) in control mice increased in the course of the experiment whereas this effect was delayed in transgenic mice (Fig 6B). In addition, the rise in the relative left ventricular weight (LVW/BW) was significantly attenuated after silencing the MR (Fig 6B). Most importantly, transgenic mice were protected from functional impairments caused by the experimentally increased cardiac afterload. Both the fractional area shortening (FAS) and the ejection fraction (EF) decreased at four weeks after TAC in controls whereas these functional parameters were almost unchanged in transgenic mice (Fig 6B). We conclude that MR silencing attenuates ventricular hypertrophy and progression to heart failure under conditions of increased cardiac afterload whereas it does not interfere with the development of cardiac fibrosis.

**Fig 7:**
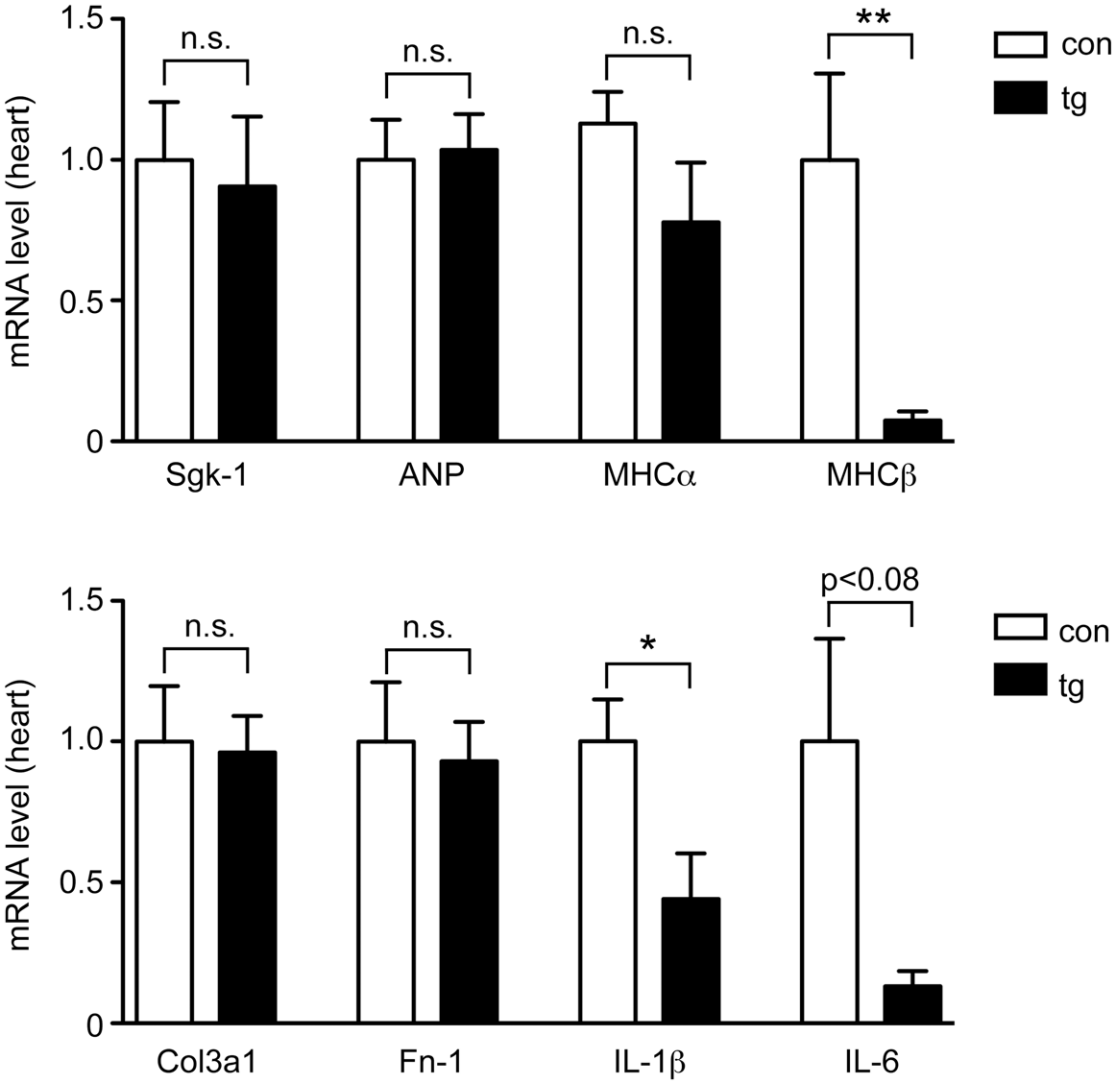
Gene expression analysis of the heart of inducible MR knock-down mice after TAC. Control (con) and transgenic (tg) mice of line B received Dox via food pellets throughout the entire duration of the experiment. TAC surgery was performed two weeks after the beginning of Dox treatment and another four weeks later the mice were sacrificed. Gene expression of Sgk-1, ANP, MHCα, MHCβ, Col3a1, Fn-1, IL-1β and IL-6 in the heart was determined by RT-QPCR. N = 5/7. Results were normalized to HPRT, and mRNA levels in control mice were arbitrarily set to 1. Values are depicted as mean ± SEM, statistical analysis was performed by unpaired two-tailed Student’s t test. n.s.: non-significant; *: p <0.05.

To support our findings we analyzed gene expression in the heart by RT-QPCR. Similar to our previous findings under unchallenged conditions (Fig 5A), Sgk-1, ANP and MHCα mRNA levels after TAC were the same in both genotypes (Fig 7). In contrast, MHCβ expression after TAC was much lower in the heart of transgenic mice than in controls, indicating that hypertrophy in response to pressure overload stress was reduced by MR silencing (Fig 7). Col3a1 and Fn-1 were both unaltered in the heart of transgenic mice after TAC (Fig 7), which is consistent with our finding that fibrosis was unaltered (Fig 6A). In contrast, the pro-inflammatory cytokines IL-1β and IL-6 were expressed at lower levels in transgenic as compared to control mice (Fig 7). Taken together, the observed selective alterations in gene expression mirror the functional changes seen in the challenged heart after MR silencing.

## Discussion

Knock-out mice are a powerful tool to analyze gene function. However, this methodology is time-consuming, costly and often results in a lethal phenotype. In contrast, the generation of knock-down mice by transgenic expression of a shRNA is much quicker, cheaper and reduces gene expression incompletely, thereby reflecting a situation often found in patients where gene function is sometimes only partially impaired [19]. Furthermore, due to the ability to induce gene silencing by application of Dox, this system is highly flexible as to the age of the mice at which the knock-down is initiated. Previously, we reported inducible insulin receptor knock-down rats which developed type II diabetes within a few days after Dox administration [21]. Here we used an improved lentiviral vector to silence the MR in transgenic mice as a tool to study kidney and heart function. Our findings indicate that the employed system allows to reduce MR levels in a variety of organs and results in physiologically meaningful changes in kidney function and the pathological heart. Furthermore, we confirmed that the system is tight, namely that there are no observable differences between transgenic and control mice in the absence of Dox. Hence the generation of inducible knock-down mice is a suitable tool to test gene function in adult animals *in vivo*, and can be achieved much quicker than it would be possible using conditional knock-out mice.

Germline disruption of the MR led to a phenotype reminiscent of pseudohypoaldosteronism type I and resulted in early postnatal lethality [4]. In contrast, selective MR ablation in the distal tubules of the kidney had only a mild effect on renal physiology [6, 7]. Loss of MR function in cardiomyocytes and macrophages provided new insight into the mechanism of heart failure but inducible approaches targeting these cell types have not been reported [9, 13, 14]. As an alternative strategy to tackle the function of the MR in adulthood, we generated transgenic mice that allow to inducibly silence the MR. Our finding that circulating levels of two major hormones involved in the RAAS, namely aldosterone and renin, were strongly increased after MR knock-down, and that this led to salt wasting, indicates that the new mouse model reflects several pathophysiological changes expected to occur after ablation of the MR. Interestingly, some genes previously considered to be targets of the MR in kidney were unaltered in Dox-treated transgenic mice, indicating that earlier findings may reflect long-term compensatory changes rather than direct effects of the MR on gene regulation.

Although the observed defects in the regulation of the RAAS as well as sodium excretion fit with our expectations, we did not find any differences in heart under unchallenged conditions with regard to gene expression and fibrosis. However, this was not fully unexpected as some MR functions only become evident under pathological conditions. This notion is supported by the analysis of cardiomyocyte-specific MR knock-out mice, which did not show any baseline effects on heart morphology and function although infarct healing was improved [9]. Indeed, the situation in inducible MR knock-down mice was comparable. When we performed TAC as a model of increased cardiac afterload, MR silencing reduced hypertrophy, diminished inflammation and interfered with progressive heart failure. The ventricular weight and the anterior wall thickness in transgenic mice increased more slowly after TAC than in control mice, which was accompanied by a lower expression of MHCβ. These findings indicate that inducible inactivation of the MR protects from cardiac hypertrophy after chronic overload pressure. In contrast, fibrosis was completely unaffected by diminished MR expression as revealed by collagen staining of the heart and RT-QPCR analysis of Col3a1 and Fn-1. Hence, at least in our model, the roles of the MR for cardiac hypertrophy and fibrosis seem to be uncoupled.

Our mouse model has both advantages and disadvantages compared to other approaches. The fact that MR downregulation occurs ubiquitously precludes any conclusions to be drawn concerning the cell type being responsible for the observed phenotype. Contrariwise, the possibility to induce downregulation of the MR in adult mice on demand allows to circumvent compensatory mechanisms, and to mimic clinical situations such as the administration of MR antagonists more closely than in constitutive knock-out mice. Therefore we expect that our inducible MR knock-down mice will become a valuable tool to address selected pathophysiological questions by complementing the already available mouse models targeting MR function.
